# Newborn Screening for Selected Disorders in Nepal: A Pilot Study

**DOI:** 10.3390/ijns5020018

**Published:** 2019-04-10

**Authors:** Arti Sharma Pandey, Suchita Joshi, Rateena Rajbhandari, Prerana Kansakar, Sadichhya Dhakal, Ralph Fingerhut

**Affiliations:** 1 Department of Biochemistry, Kathmandu Medical College (Basic Sciences), Duwakot, Bhaktapur 44802, Nepal; 2 Department of Pediatrics, Patan Academy of Health Sciences, Patan, Lalitpur 44700, Nepal; 3 Intern, Kathmandu Medical College and Teaching Hospital, Sinamangal, Kathmandu 44600, Nepal; 4 Newborn Screening Laboratory, 8032 Zurich, Switzerland

**Keywords:** newborn screening, Nepal, congenital hypothyroidism, cystic fibrosis, hemoglobinopathies

## Abstract

The prevalence of metabolic disorders in Nepal is yet unknown, although many case reports occur in literature. Heel-prick blood samples from newborns were collected on Dried Blood Spot (DBS) collection cards and tested through Tandem Mass Spectroscopy and fluorescence assays for disorders included in the Swiss neonatal screening program; two cases of hypothyroidism and one case of cystic fibrosis were identified. Thyroid stimulating hormone (TSH), immuoreactive trypsinogen (IRT), hydroxyprogesterone (OHP), tyrosine (Tyr), and octanoylcarnitine (C8) showed significant differences with gestation age. Most of the parameters were positively correlated with each other except galactose, galactose 1 phosphate uridyl transferase (GALT), and biotinidase. First and ninety-ninth percentiles in the Nepalese newborns were found to be different when compared with the Swiss newborns. Congenital hypothyroidism and cystic fibrosis are candidates to be considered for a newborn screening program in Nepal. Differences between the Nepalese and Swiss newborns in parametric values that change with gestation age can be attributed to a higher survival rate of pre-term babies in Switzerland. Others could be explained in part by early and exclusive breastfeeding in Nepalese newborns.

## 1. Introduction

Inborn Errors of Metabolism (IEMs) are increasingly recognized as representing examples of complex gene nutrient interactions leading to complex disease [1]. The classification of Disease version 11 (ICD 11) lists IEMs under Metabolic Diseases, which itself is a sub-class of Endocrine Nutritional or metabolic diseases [2].

Nepal is a small country which can geographically be divided into the flatlands in the south, known as the *terai* belt, the hilly regions in the center, and the high mountains in the North. The country is home to people of 125 castes and ethnicities, as reported in 2011: *Chhetris* constitute the largest caste, followed by *Brahmins*, *Magars* and *Tharus* [3]. The *Tharus* largely inhabit the southern *terai* belt and are reported to be the aboriginal population of Nepal, with the rest having migrated from South-West China and India. According to race, the population can be divided into four major groups [4]: Aryans, Mongoloids, Newars (Tibeto-Burmans), and Tharus. Many different castes belong to these broader races, which originate from the areas these populations migrated from. The prevalence of IEMs in Nepal is not known yet. Many metabolic disorders have been reported in Nepal individually, spanning carbohydrate and lipid metabolism, lysosomal storage disease, congenital hypothyroidism, and hemoglobinopathies [5,6,7,8,9]. A urinary screening study on samples from mentally retarded children utilizing chemical methods reports the detection of mucopolysaccharidosis and organic acidurias, though not confirmed through enzymatic or genetic diagnostic tests [10]. Although consanguinity has become quite uncommon compared to older times in Nepal, yet the caste system is still adhered to by a large proportion of the population during marriages: 21.6% of Nepalese do not accept to marry their son/daughter outside of their caste [4]. A strong example of this is seen in the alarmingly high incidence of hemoglobinopathies amongst the *Tharu* populations of the western *terai* region of Nepal. In a study on anemia in pregnant women, of the 2% of women of reproductive age that were found to have sickle cell trait, 94.7% belonged to the *Tharu* community [11]. Another hospital based study identified sickle cell disease and thalassemias as the most commonly occurring hemoglobinopathy, with a majority of these (37.6%) affecting the *Tharu* community [12]. A genetic analysis of thalassemias amongst this population in 1991 [5] found the majority of the *Tharus* with the α−/α− genotype with an α− gene frequency of 0.8. This genotype was found to be most often due to the deletion of 3.7 kb leading to a loss of one α globin gene with a frequency of 0.63 as compared to 0.05% among a population living in malaria free uplands [13].

IEMs as a class of disorders are difficult to diagnose and require specific training and expertise for recognition and therapy. A screening panel or diagnostic testing for IEMs is non-existent in Nepal. The clinical presentation of an IEM in a neonate is almost always non-specific [14] and most often comprises of poor suckling, hypotonia, tachypnea or respiratory distress, vomiting, and seizures. IEMs such as *O*-glycosylation disorders, cholesterol synthesis, and amino acid synthesis disorders, among others, can result in true irreversible malformations in the antenatal period [15]. Other metabolic disorders like fatty acid oxidation disorders, respiratory chain disorders, or carnitine uptake defects manifest as cardiac disease [15]. The most common underlying causes of neonatal death in Nepal have been reported to be respiratory and cardiovascular disorders of the perinatal period (31%); and complications of pregnancy, labor, and delivery (31%) [16]. Congenital malformations and disorders related to length of gestation and fetal growth account for 7% and 2% of neonatal deaths, respectively. It is logical to assume that some cases of neonatal deaths recorded in the health survey of Nepal [16] might have been due to IEMs.

This pilot study targeting 5000 samples was a collaboration between Kathmandu Medical College and Teaching Hospital, Nepal, and the Newborn Screening Laboratory, in Zurich, Switzerland, to identify the IEMs that might be prevalent in the Nepalese children from amongst the ones included in the Swiss newborn screening program.

The current statistics on Nepal report the infant mortality rate in Nepal to be 29.6 per 1000 live births [16], while that of Switzerland is 3.6 per 1000 live births [17]. Switzerland started newborn screening in 1965 with phenylketonuria and expanded to the current panel for national newborn screening, which has added congenital adrenal hyperplasia (CAH), medium chain acyl CoA dehydrogenase deficiency (MCAD), cystic fibrosis (CF), galactosemia, glutaric acidemia type 1 (GA 1), maple syrup urine disease (MSUD), and biotinidase deficiency [18] to the program. The Newborn Screening Laboratory in Zurich utilizes automated tandem mass spectrometry and fluorometric immunoassays for measurements of analytes.

## 2. Materials and Methods

Ethical clearance for collection of heel-prick blood samples from neonates was obtained from the Nepal Health Research Council, Nepal, registration number 206/2013 on 26 January, 2014. Heel-prick blood samples were collected from all neonates born at Kathmandu Medical College and Teaching Hospital in Kathmandu district, Patan Academy of Health Sciences in Patan district, and the Missions Hospital in Palpa district. Samples were collected on Dried Blood Spot (DBS) collection cards after obtaining informed consent from the parents. The cards were air dried in shade and kept in a cool and dark closet until they were shipped to Zurich. Care was taken not to expose the cards to temperature extremes nor moisture during collection from the participating institutions. Demographic data was entered through EpiData, after which the cards were shipped to the National Newborn Screening laboratory in Zurich, Switzerland, once a month.

The samples were tested for metabolites that are tested as a part of national neonatal screening program at the Newborn Screening Laboratory at Zurich, Switzerland. The values from the Swiss neonatal screening during the same time were used for comparison with the Nepalese values. The analytes measured were thyroid stimulating hormone (TSH), total galactose (tGal), galactose 1 phosphate uridyl transferase (GALT), 17 α hydroxyprogesterone (OHP), immuoreactive trypsinogen (IRT), phenylalanine (Phe), tyrosine (Tyr), leucine (Leu), isoleucine (Ile), valine (Val), glutarylcarnitine (C5DC), and octanoylcarnitine (C8).

Quantitative estimations of tGal, GALT, OHP, TSH, and IRT were carried out using genetic screening processor (GSP) neonatal screening kits through fluorescence assays or fluorescence immunoassays on the GSP Analyzer by PerkinElmer (Turku, Finland). Controls provided by GSP were run along with internal controls created for the lab using sheep blood for each assay to ensure validity of results. Simultaneous quantitative analysis of Phe, Tyr, Leu, Ile, Val, C5DC, and C8 were carried out through tandem mass spectrometry on Waters XEVO-TQD (Milford, CT, USA), using the NeoBase Non-derivatized MSMS Kit from Perkin-Elmer (Turku, Finland). Samples showing high values were confirmed through mutational analysis.

Statistical analysis was done using the “R” software [19]. Non-parametric analysis was done using the Wilcox rank sum test from the basic R package “stats”, using a confidence interval of 95% and α = 0.05. Pearson’s correlation heat map was created using the “corrplot” package in R [20]. Distribution was assessed for all analytes by creating a histogram matrix with “ggplot2” [21].

## 3. Results

The three tertiary hospital sites of sample collection receive patients from the districts of Kathmandu, Bhaktapur, Lalitpur, Palpa, Gulmi, Syangja, and Arghakhachi, with a combined population of 354,514, 13.4% of the total population of Nepal [22]. Fifty-five percent of the samples were collected on day one of life. The population covered was divided according to the anthropological divisions by Dor Bahadur Bista [23] into people of Indo-Aryan, Mongol, Newar, and the indigenous Tharu origins. Accordingly, our study population comprised 78% Aryans, 11% Newars, 10% Mongols, and 0.5% Tharus. A total of 4360 samples had complete data for statistical analysis. The Tharu representation in the population studied was inadequate compared to the national proportion of Tharus (6.2%) [3]. The maximum number of underweight infants, both term (≥36 weeks) as well as pre-term (<36 weeks), belonged to the Aryans, while most pre-term deliveries occurred amongst Newars (3.9%).

### 3.1. Birth Weight Statistics

Birth weight was moderately positively correlated with the infant’s gestational age. Only 0.9% of the babies weighed less than 1902 g, the 1st percentile, while 1.1% weighed more than 3957 g, the 99th percentile. Four hundred and seventy (11.5%) infants were of low birth weight (<2500 g), of whom five were of very low birth weight (<1500 g) and one was extremely low birth weight (<1000 g). All analytes in the low birth weight infants were within the 99th percentile. The birth weight for male babies (mean = 3022 g, sd = 450) was significantly higher (*p* < 0.001) than female babies (mean = 2924 g, sd = 429). There was no significant difference between the gestational ages of males and females (*p* = 0.1367).

### 3.2. Overview of Analytes

**Figure 1:**
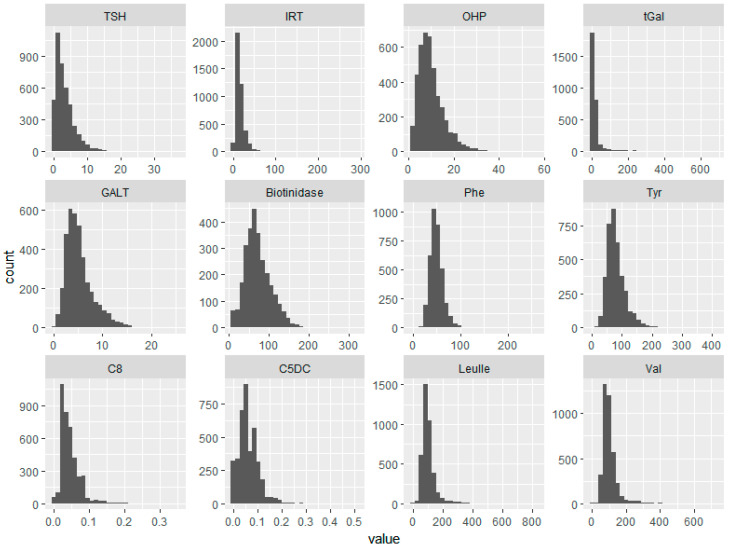
The distribution curves of all analytes from 4360 sample values. TSH—thyroid stimulating hormone, IRT—immunoreactive trypsinogen, OHP—17-α-hydroxy progesterone, tGal—total galactose, GALT—galactose-1-phosphate uridyl transferase, Phe—phenylalanine, Tyr—tyrosine, C8—octanoylcarnitine, C5DC—glutarylcarnitine, Leu/Ile—leucine/isoleucine, Val—valine. Units of measurement are as in Table 1.

The analyte values exhibited exponential, log normal, or γ (Figure 1) distributions. The mean, standard deviation, and percentiles of all measured analytes are as shown in Table 1, with Swiss percentiles for comparison. The 1st percentile values of all analytes were either comparable or lower than the corresponding values from the Swiss population, while the 99th percentile values in the Nepalese samples were comparable or higher. The activities of GALT and biotinidase were lower than the corresponding Swiss percentiles, while the IRT levels were higher.

**Figure 2:**
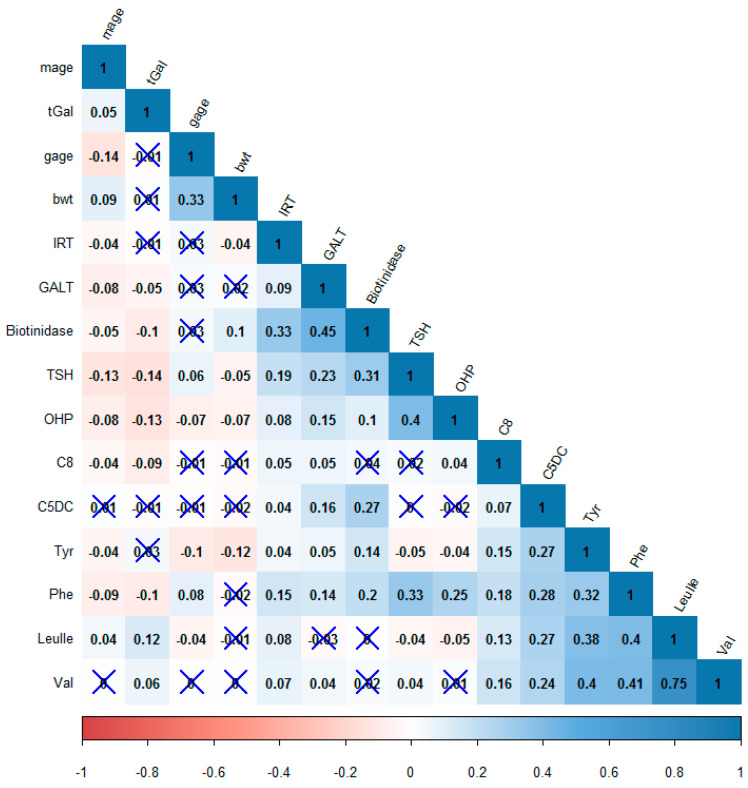
A Pearson’s correlation matrix heat map of the parameters measured in the study. Red—negative correlation, Blue—positive correlation. Correlations with insignificant *p*-values are marked with an “X”. mage = Mother’s age, gage = gestation age, and bwt = birth weight.

A Pearson’s correlation matrix was constructed in order to identify patterns amongst the variables (Figure 2), where *r* ≥ 0.5 and *r* = 0.3–0.5 was considered as strong or moderate correlations, respectively. Excluding demographics, the strongest positive correlation was between Val and Leu/Ile (*r* = 0.75), while all negative correlations were weak (*r* < 0.3). TSH was moderately positively correlated with OHP, Phe, and biotidinase. All amino acids were moderately positively correlated to each other, as were all measured enzymes.

### 3.3. Gestational Age

The data was divided into two subgroups based upon gestation age into <36 weeks and ≥36 weeks categories to examine the effect of gestational age on metabolic analytes. Non-parametric analysis was carried out on the two subgroups using the Wilcox rank sum test with continuity correction (Table 2). Birth weight and Tyr in the two groups showed high differences, which was statistically significant (*p* < 0.001). TSH, IRT, OHP, and C8 also differed by small but significant amounts.

### 3.4. Biotinidase

Biotinidase activity was significantly higher (*p* < 0.001) in females but did not vary with gestational age. There was a strong positive correlation between biotinidase activity and the other two enzymes measured in this study, IRT (*r* = 0.33) and GALT (*r* = 0.45) (Figure 2). The cutoff for a profound biotin deficiency, taken as 10% of mean [24] was 7.27U, was found in only one male infant. The infant’s parents were contacted in 2018, and the child was found to be developing normally.

### 3.5. IRT

IRT values were higher compared to Swiss percentiles, but quite similar when only infants more than 24 h of age at the time of sample collection were considered. Forty-two samples had a value higher than 63U, the 99th percentile. One case of cystic fibrosis was detected based upon a very high IRT value of 290 μg/L. A confirmatory test using the Cystic fibrosis Strip Assay verified the disorder to be due to a homozygous ΔF508 deletion, giving an incidence of 1:4360. IRT levels were mildly but significantly negatively correlated to birth weight. It was moderately positively correlated (*p* < 0.001) with TSH, Phe, and biotinidase levels.

### 3.6. OHP

There was a small but significant negative correlation between OHP levels and birth weight (*r* = −0.06, *p* < 0.01), as well as OHP and gestation age (*r* = −0.07, *p* < 0.01) (Figure 2). Females had significantly lower (*p* < 0.001) OHP levels compared to males. Forty-one infants had values higher than the 99th percentile, of which three were of low gestational age as well as underweight, one was low gestational age only, and five were underweight only.

### 3.7. TSH

TSH values were observed to be negatively correlated to mothers’ age (*p* < 0.001, Pearson’s *r* = −0.13) and positively correlated to gestational age (*p* < 0.001, Pearson’s *r* = 0.064) (Table 1). There was a mild but significant negative correlation of TSH with birth weight (*p* < 0.001) (Figure 2). Forty-three samples had TSH values greater than the 99th percentile (13 mIU/L), three of whom were from Kathmandu, the rest being from areas of west Nepal. All percentiles were higher than Swiss values. Two cases of congenital hypothyroidism were detected (TSH values 36 and 230 mIU/L), and the parents were informed immediately.

### 3.8. Galactose

tGal and GALT showed a mild but significant negative correlation (*r* = −0.05, *p* = −0.01) (Figure 2) with each other. There was no significant difference between mean tGal or GALT according to gestational age (Table 1). tGal was negatively correlated to measured analytes except Tyr, Val, and Leu/Ile, while GALT was positively correlated with all analytes except Leu/Ile (Figure 2).

### 3.9. Amino Acids

The amino acid values did not differ significantly with gestational age. Abnormally high values of Val and Leu/Ile were detected in three infants, which were found to be normal after a confirmatory test. Tyr values were lower than the Swiss levels, despite the fact that 55% of the samples had been collected within 24 h of birth. The distribution curves of Phe and Tyr in the Nepalese newborns were close to normal (Figure 1).

### 3.10. Carnitines

C5DC and C8 levels were comparable to the Swiss values. The infants with the highest values of C5DC and C8 also had very high values of Val, which were 532 and 491 µmol/L, respectively.

## 4. Discussion

The national census of Nepal, 2011 [3], reports 125 castes and ethnic groups, of which 42% belong to the Aryans, 15.8% to Mongolians, 6.2% to *Tharus*, and 5.6% to Newars, among other minor groups which can be assumed to be of Aryan origin till more data becomes available. The Tharus were underrepresented in the presented study, as most *Tharus* reside in the *terai* belt in the southern part of the country. All other ethnicities live in large numbers in the districts of Kathmandu, Patan, and Palpa.

The proportion of children with low birth weight was lower than the reported UNICEF values of 18% for Nepal (UNICEF, 2009–2015) [25]. In Nepal, early initiation of breast feeding (within 1 h of birth) occurs in 49% of births, and 57% of infants are exclusively breastfed for 6 months.

Plasma branched chain amino acid (BCAA) concentrations in mammals tend to be directly proportional to protein intake. The concentrations of BCAAs were very strongly positively correlated (Figure 2) with each other. The first two steps of transamination and dehydrogenation in the catabolism of BCAAs are common to all BCAAs and account for the remarkable correlation among the plasma levels of the three BCAAs in a variety of situations, [26] as was also observed in this study. The moderate positive correlations of enzyme activities to each other are likely due to the conditions of shipping, influencing enzyme activities similarly. A positive correlation between TSH and OHP has been reported in another study, which attributes it to stress in the neonate signified by raised OHP that, in turn, results in raised TSH [27]. Although a correlation between Phe and TSH has not been reported so far, the below optimal activities of phenylalanine hydroxylase in newborns [28], considered together with the physiological neonatal surge of TSH during the one or two days after birth [29], can provide an explanation. Newborns closer to the time of birth are likely to be experiencing both a surge of TSH, as well as a reduced phenylalanine hydroxylase activity, the latter causing plasma Phe levels to be higher. A correlation between TSH and biotinidase has not been reported in literature before. Individual analytes are discussed below.

### 4.1.TSH

This study reports an incidence of congenital hypothyroidism (CH) of 1:2500, compared to a reported incidence of 1:2000 in a seven-year study of newborn screening [30]. Various states in India have reported an incidence of CH ranging from 1:22 to 1:13,426 [31]. Both children detected with CH through this study are now on hormonal therapy and show normal development except delayed speech in one of them. Iodine deficiency can also result in transient hypothyroidism and increase false positives. According to the 2016 Demographic and Health survey [16], 95% children in Nepal live in households consuming iodized salt. A study conducted in the remote areas of Shree Antu and Ranke of Eastern Nepal amongst children 6–12 years of age found 33.6% children to have insufficient iodine excretion [32]. A 41% prevalence of subclinical or overt hypothyroidism in pregnant women has been reported in a hospital based study in Western Nepal [33]. Another study on iodine deficiency and hypothyroidism amongst pregnant women found 3.2% of the cases positive for antibodies to thyroid peroxidase and 18.5% of the women to have inadequate levels (<150 µg/L) of iodine [34]. In a study of children diagnosed with congenital hypothyroidism [9], thyroid agenesis, dyshormogenesis and ectopic thyroid were the most common causes of low thyroid hormones. In lieu of autoimmune thyroid disorders, as well as iodine deficiency in mothers, any screening for thyroid disorders in neonates in Nepal will require simultaneous assessment of the mother’s thyroid status and continuous monitoring of the child until developmental landmarks in the baby have been met.

### 4.2. OHP

The inverse relationship of OHP concentrations with gestational age and birth weight is well-documented [35] and was also seen in the Nepalese samples (Figure 2). However, despite a lower birth weight of female infants, the OHP was lower compared to males. Similar results have been obtained for two million babies screened for CAH, where the female babies were found to have significantly lower birth weights and lower OHP values; however, there was no significant difference between the numbers of males and females who had a confirmed diagnosis of CAH [36]. Higher values of OHP compared to the Swiss values was most likely due to an earlier collection of heel-prick samples: 55% collections were done during the first day of life. One case of a 12-year-old boy with CAH confirmed to be due to 11 β hydroxylase deficiency has been reported in Nepal [37]. People belonging to the “intersex” community of Nepal have been documented [38], several of whom were born females and developed male characteristics during puberty, and could possibly be undiagnosed cases of congenital adrenal hyperplasia. Because of the stigma attached to being born with ambiguous genitalia, along with the preference for a male child, CAH is very likely to be an underreported disorder in Nepal.

### 4.3. IRT

In comparison with the Swiss samples, the percentiles of IRT in the Nepalese infants is much higher, considering the time samples were exposed to possible humidity and room temperature over the period of shipping. High IRT levels are not always the result of cystic fibrosis transmembrane regulator (CFTR) mutation [39]. The probability that a newborn with high levels of IRT is a carrier for one of the mutations causing CF, however, increases with an increase in IRT levels [40]. Non-CF causes of an elevated IRT include dried blood spot samples contaminated with meconium, neonatal stress, respiratory distress, hypoglycemia, congenital abnormalities, congenital infections, bowel atresias etc. [39]. There have been no reports of cystic fibrosis in Nepal in the literature, and this study is the first to report one. The baby diagnosed with the condition belonged to a poor family in the hills, and did not survive beyond 3 months. As the early clinical manifestations of CF in infants are mainly pulmonological and there is also a lack of diagnostics at hospitals in Nepal for CF other than through clinical presentation, many cases of CF probably remain undiagnosed.

### 4.4. Galactose, GALT, and Biotidinase

Total galactose, GALT, and biotinidase levels decline at temperatures around 35 °C as well as in the presence of humidity. The collections had been done throughout the year and sent in batches to Zurich. Individual batches, hence, might have differed in losses of activity of levels in the dried blood spots; but the range of values still provides a good estimate of the percentiles. The transport was in dry, sealed envelopes and care was taken not to expose the cards to high temperature and humidity during collection. Higher IRT and TSH values compared to the Swiss samples, which are shipped to the laboratory within a week of collection, suggests that the exposure to humidity was very limited. The measured GALT and biotinidase activities in the Nepalese samples might be lower than it would have been in fresh samples, and probably due to high temperature, longer storage and transportation time lapse before analysis. The higher levels of galactose in the Swiss samples compared to the Nepalese samples could be due to various reasons. One consideration is the proportion of babies that are exclusively breast fed. Galactose is cleared by the neonatal liver much faster than glucose [41] and it can be hypothesized that the conversion of galactose to glucose [42] in breast fed infants might be higher. Breast fed infants have been shown to have higher levels of glucose compared to lactose based formula fed babies [43]. Another explanation for differences in the Nepalese and Swiss galactose levels is differences in the expression of galactose metabolizing enzymes in the two populations, which would be interesting to investigate. No instances of galactosemia in Nepal have been reported in the literature.

### 4.5. Amino Acids

The measured amino acids in the Nepalese infants were lower than the percentiles in the Swiss infants with the exception of phenylalanine. The assumption about the Swiss infants being more formula fed can also provide some basis for this difference as Infant formula contains higher protein compared to human milk [44]. Exclusively breastfed infants have been found to have much lower levels of Leu, Ile, Tyr, and Val [43]. Breast milk also empties faster than formula milk, so that amino acid levels in blood stay elevated for longer in formula-fed infants [45]. A clinically diagnosed case of alkaptonuria [6] has been reported in a 52-year-old male in Nepal. No cases of branched chain ketoacidurias or Hartnup disease have been reported from Nepal. No cases of biotinidase deficiency, MCAD deficiency, or glutaricacidemia have been reported from Nepal.

### 4.6. Implications for Nepal

This is the first large scale study for detection of selected metabolites in the heel-prick blood samples from newborns in Nepal. The study lays the foundation of a newborn screening program in Nepal, while providing the first reference ranges for various metabolites for neonates. Genetic disorders in Nepal are mostly reported through individual cases by newspapers, as case reports in peer reviewed journals and sometimes through organized studies. As a result, hemoglobinopathies have been recognized as rampant amongst the *Tharu* population of Nepal. There are also organizations formed by affected individuals’ parents for providing care and lobbying with the government for provision of healthcare for these rare diseases. Some such self-help groups are the Nepal Thalassemia Society (www.nepalts.org.uk), Muscular Dystrophy Foundation-Nepal (www.mdfnepal.org), and Cerebral Palsy Nepal (www.cpnepal.org). A population based study for sickle cell anemia among the *Tharus* is currently underway in the district of Bardiya in the *terai* region of Nepal [46]. Metabolic pathways enzyme deficiencies and lysosomal enzyme deficiency reports are largely anecdotal. Nevertheless, a case for including lysosomal storage diseases and hemoglobinopathies in any screening program can be made for Nepal, based upon literature reports [47,48].

Hypothyroidism has been noted amongst children in Nepal largely because of a traditional iodine deficiency amongst the mountain regions. This has been addressed over the years by the Nepalese government by providing salt fortified with iodine to all areas of the country. Based upon our study, it is worthwhile to include hypothyroidism in a screening program, as this will also identify hypothyroidism due to iodine deficiency, which can be rectified immediately. With a current prevalence of cystic fibrosis of 1:4360, it would also be worthwhile to include this disorder in any screening program.

## 5. Limitations of Study

The main limitation of the study is its small sample size that excludes people from the *terai* as well as the high mountains. The percentiles reported in this pilot study are bound to change with a larger study spread over the whole nation, while also identifying disorders that might be more relevant to Nepal for screening.

## Figures and Tables

**Table 1:** Measured parameters in the Nepalese population in comparison with the 1st and 99th percentiles in the Swiss population.

Variable	Range	mean	SD	Percentiles			
				Nepalese		Swiss	
				1st	99th	1st	99th
Gestational age	27–44	38.99	1.68	33	42	*	*
Birth weight (g)	815–5515	2975.82	442.70	1902.2	3957.25	1195	4420
Mother’s age (years)	15–45	24.95	4.61	17	39	*	*
TSH (mIU/mL)	0–230	3.33	2.84	0.1	13.1	0.30	6.30
IRT (μg/L)	0.1–290.9	16.81	12.67	3	63.3	5.8	49.0
OHP (nmol/L)	1–55.5	10.08	5.68	2	28.9	1.6	14.5
tGal (μmol/L)	0–680.4	24.23	56.56	0	312.87	0	532.7
GALT (U/dl)	0–25.2	5.30	2.84	1.1	14.3	5.9	22.7
Biotinidase (U)	6.8–315.8	72.72	32.18	11	157.50	100.2	353.9
Phe (μmol/L)	0–259.6	50.39	14.57	25.08	90.63	25.7	69.2
Tyr (μmol/L)	6.95–420.73	80.35	31.76	31.68	182.67	28.5	206.1
C8 (μmol/L)	0–4.03	0.050	0.07	0	0.17	0.00	0.14
C5DC (μmol/L)	0–3.62	0.064	0.07	0	0.21	0.02	0.21
Leu/Ile (μmol/L)	0–1499.16	106.43	55.60	42.36	312.53	71.5	255.1
Val (μmol/L)	0–2578.74	104.63	67.53	44.35	320.03	56.6	245.9

* Mother’s age is not recorded in the Swiss New Born Screening program. Gestational age from Swiss data was not available.

**Table 2:** Measured parameters comparison between pre-term (<36 weeks) and term (≥36 weeks) infants.

Variable	W	Difference in Location	*p*-Value	Confidence Interval (95%)	Mean	
					≥36 weeks	<36 weeks
Birth weight (g)	387,580	530	<0.001	450, 615	2993.7	2449.09
Mother’s age (years)	173,840	−1.00	0.0147	−2.16, 0.00003	24.91	26.1
TSH (mIU/L)	254,590	0.40	0.017	−0.099, 0.799	3.26	2.88
IRT (μg/L)	249,790	1.299	0.031	−0.1, 2.499	16.58	15.50
OHP (nmol/L)	199,820	−0.999	0.042	−1.9, 0	9.94	11.42
tGal (μmol/L)	111,950	0.599	0.358	−0.699, 2.20	24.79	18.03
GALT (U)	182,070	0.099	0.654	−0.299, 0.499	5.04	4.71
Biotinidase (U)	92,178	3.90	0.188	−1.90, −9.99	68.83	63.52
Phe (μmol/L)	171,240	1.01	0.425	−1.48, 3.52	50.05	48.83
Tyr (μmol/L)	141,630	−12.99	<0.001	−20.23, −6.02	79.09	94.60
C8 (μmol/L)	170,950	−0.01	0.007	−0.01, −0.00005	0.05	0.06
C5DC (μmol/L)	197,350	0	0.813	−0.01, 0.01	0.062	0.07
Leu/Ile (μmol/L)	180,800	−5.86	0.080	−12.51, 0.72	106.45	114.83
Val (μmol/L)	188,490	−3.27	0.290	−9.53, 2.86	104.67	107.48

W—Wilcoxon rank sum test statistic.
